# Sickness absence and disability pension due to otoaudiological diagnoses: risk of premature death – a nationwide prospective cohort study

**DOI:** 10.1186/1471-2458-14-137

**Published:** 2014-02-08

**Authors:** Emilie Friberg, Ulf Rosenhall, Kristina Alexanderson

**Affiliations:** 1 Department of Clinical Neuroscience, Division of Insurance Medicine, Karolinska Institutet, Stockholm SE 171 77, Sweden; 2 Center for Hearing and Communication Research, Department of Clinical Neuroscience, Karolinska Institutet, Stockholm, Sweden; 3 Department of Audiology and Neurotology, Karolinska University Hospital, Stockholm, Sweden

**Keywords:** Hearing diagnoses, Sick-leave, Mortality

## Abstract

**Background:**

It is estimated that hearing difficulties will be one of the top ten leading burdens of disease by 2030. Knowledge of mortality among individuals on sick leave or disability pension due to hearing diagnoses is virtually non-existent. We aimed prospectively to examine the associations of diagnosis-specific sick leave and disability pension due to different otoaudiological diagnoses with risks of all-cause and cause-specific mortality.

**Methods:**

A cohort, based on Swedish registry data, including all 5 248 672 individuals living in Sweden in 2005, aged 20–64, and not on old-age pension, was followed through 2010. Otoaudiological diagnoses were placed in the following categories: otological, hearing, vertigo, and tinnitus. Hazard ratios (HRs) with 95% confidence intervals (CIs) were estimated using Cox proportional hazard models; individuals on sick leave or disability pension due to different otoaudiological diagnoses during 2005 were compared with those not on sick leave or disability pension.

**Results:**

In multivariable models, individuals with sickness absence due to otoaudiological diagnoses showed a lower risk of mortality, while individuals on disability pension due to otoaudiological diagnoses showed a 14% (95% CI 1-29%) increased risk of mortality, compared with individuals not on sick leave or disability pension. The risk increase among individuals on disability pension was largely attributable to otological (HR 1.56; 95% CI = 1.04-2.33) and hearing diagnoses (HR 1.20; 95% CI = 1.00-1.43).

**Conclusion:**

This large nationwide population-based cohort study suggests an increased risk of mortality among individuals on disability pension due to otoaudiological diagnoses.

## Background

Hearing difficulties are a serious public health problem [1-4]. It has been estimated that they will become one of the top ten leading burdens of disease by 2030 [3]. Hearing difficulties are not only a problem among the elderly, but also a common problem in the working-age population [2,5,6]. Any influence that hearing difficulties might have on mortality has, however, been only sparsely examined.

All-cause sick leave has previously been found to be associated with increased mortality [7-13], as too has all-cause disability pension [14-18]. However, knowledge of diagnosis-specific sick leave and diagnosis-specific disability pension is very scarce. In particular, knowledge of sick leave and disability pension resulting from specific otoaudiological diagnoses is very limited due to the small sizes of the groups involved [19-21]. In two recent prospective studies, however, we have shown that sick leave due to an otoaudiological diagnosis is associated with an increased risk of disability pension in comparison with sick leave due to other diagnoses [22,23].

The aims of this study were, for the first time, to assess whether individuals with sick leave and disability pension due to different otoaudiological diagnoses are associated with an increased risk of premature death (both total and cause-specific) compared with individuals with no sick leave or disability pension, and also to assess whether any associations found differed according to age or gender. Thus we performed a prospective study of the Swedish population of working age, 2005-2010.

## Methods

The study cohort consisted of all 5 248 672 individuals living in Sweden throughout the year 2005, who were aged 20–64, and who were not on old-age pension (29 025).

We used the following information from three different national registers:

– →From Statistics Sweden’s Longitudinal Integration Database for Health Insurance and Labor Market Studies (with Swedish acronym LISA), we obtained annual information on several potentially relevant factors: sex, age, education, family situation, type of living area, country of birth, and old-age pension. Information from LISA was used both to define the cohort and for follow-up.

– →From the National Social Insurance Agency Register, we retrieved information on sick-leave benefits paid by the Agency and associated case diagnoses, on disability pensions and associated case diagnoses, and on old-age pensions.

– →From the National Board of Health and Welfare, we acquired information on the number of days hospitalized during 2000–2005, on visits to specialized outpatient clinics, and on dates and causes of death.

The three registers all hold information at individual level, which permits record linkage using the unique personal identification numbers assigned to all residents in Sweden. In Sweden, a sickness certificate issued by a physician is required to obtain sickness benefit after the 7^th^ day of a sick-leave spell. All individuals in Sweden can be granted disability pension if they have permanent work incapacity due to disease or injury. Moreover, all individuals in Sweden with earnings are covered by the same public sickness insurance (which covers the employed, the self-employed, job seekers, students, individuals on parental leave, etc.). For most employed individuals, sickness benefit is paid by the Social Insurance Agency from the 15^th^ day. For most people, 65 is the age for old-age pension, but it can be taken earlier. Sweden has a high employment rate, among both women and men, and also among people of higher ages.

Migration was assessed as not having lived in Sweden for two consecutive years (without having a date of death). The date of migration was set at December 31 for the first missing year; in total, 66 592 individuals were censored for this reason.

We calculated person-time of follow-up for each individual from January 1, 2006 to the date of death, migration, or end of follow-up (December 31, 2010), whichever came first. Hazard ratios (HRs) for all-cause and cause-specific mortality and 95% confidence intervals (CIs) were estimated in Cox proportional hazards models, using person-time at follow-up as the underlying time scale. The data conformed to the proportional hazards assumption, as was established by graphic inspection of survival function versus survival time, and by maximum likelihood tests on time-dependent covariates in each model.

Both sick-leave and disability-pension diagnoses were classified according to the International Statistical Classification of Diseases and Related Health Problems, version 10 (ICD-10) [24]. Exposure was categorized as either: 1) having at least one prevalent (i.e., on-going or new) sick-leave or disability pension during 2005 due to an otoaudiological diagnosis (ICD-10 chapter VIII, H60-H95); or 2) having at least one prevalent sick-leave spell or disability pension during 2005 due to a non-otoaudiological diagnosis (or a non-registered diagnosis), or 3) having no sick-leave and no disability pension compensated for by the Social Insurance Agency during 2005 (reference category). All individuals with an otoaudiological diagnosis for a sick-leave spell or disability pension were included in the otoaudiological categories, even if they had also taken non-otoaudiological sick leave or disability pension during 2005.

Moreover, for the detailed analyses, the otoaudiological diagnosis category was broken down, with four subcategories replacing the single otoaudiological category in the models. The subcategories were mainly: “otological” (H60-H75, H80, H92, H94, H95), “hearing” (H83, H90, H91), “vertigo” (H81, H82), and “tinnitus” (H93). Individuals who had more than one sick-leave spell due to two different otoaudiological diagnoses were included in both subcategories.

We performed crude, age- and sex-adjusted, multivariable analyses, with further adjustment to include “morbidity”, operationalized in terms of number of hospitalization days during the last five years (2000–2005). In the main analysis of all-cause mortality, we adjusted for age (20–34, 35–44, 45–54, 55–64), sex (female, male), family situation (married/cohabitant, married/cohabitant with children, single, single with child, child living with parent), type of living area (larger cities, medium-sized cities, smaller places), country of birth (Sweden, Nordic countries, Europe, other part of the world), educational level (0–9, 10-12, >12 years), and hospitalization days (inpatient) 2000–2005 (total days: 0, 1–4, >4). Further, in the analysis of cause-specific death, we included the risk of death due to circulatory disease (ICD10; I00-I99) or cancer (ICD10; C00-C97, D00-D48), with adjustments made for number of visits to specialized outpatient clinics in 2001–2005 with a diagnosis within the same ICD10 chapter as the cause of death. Moreover, an analysis stratified by age and sex was performed, since both age and sex have been reported to influence sick leave and disability pension as well as mortality.

All the statistical analyses were performed using SAS software (version 9.2; SAS Institute, Cary, NC). The project has been evaluated and approved by the Regional Ethical Review Board of Karolinska Institutet, Stockholm, Sweden.

## Results

During the mean follow-up period of 4.9 years, 72 112 people died due to all causes among the cohort of 5 248 672 individuals (25 846 504 person years). The mean age of death due to all causes was 56.6 (±9.8). In Table 1, the baseline characteristics of the cohort, in different categories of sick-leave diagnoses, and with different disability-pension diagnoses, are presented. Individuals with at least one sick-leave spell during 2005 due to an otoaudiological diagnosis were on average older, more often married/cohabiting, born in a country other than Sweden, and had been hospitalized, and also lived less often in a large city compared with individuals with no sick-leave spells or disability pension in 2005. Individuals with a hearing or tinnitus sick-leave diagnosis had on average a higher educational level than those with no sick-leave benefit or disability pension in 2005. Individuals on disability pension due to an otoaudiological diagnosis during 2005 were, on average, older, more hospitalized, born in a country other than Sweden (especially individuals with an otological diagnosis), and less likely to be married/cohabiting, and had, on average, a lower education, and lived less often in a large city than individuals with no sick-leave spells or disability pension in 2005.

**Table 1:** Baseline characteristics, 2005

		**Sick leave**						**Disability pension**					
			**Sick leave due to otoaudiological diagnoses**						**Disability pension due to otoaudiological diagnoses**				
	**Reference group**^**a**^	**Other diagnoses**	**All**	**Otological**^**b**^	**Hearing**^**c**^	**Vertigo**^**d**^	**Tinnitus**^**e**^	**Other diagnoses**	**All**	**Otological**^**b**^	**Hearing**^**c**^	**Vertigo**^**d**^	**Tinnitus**^**e**^
N	4 099 925	701 913	5 319	1 407	1 186	1 629	1 130	479 178	12 549	835	6 738	2 092	5 267
Women (%)	46.12	61.87	59.90	60.55	66.3	63.54	47.43	60.05	59.58	67.66	61.22	66.59	52.31
Age (years)	40.7	44.8	49.6	45.4	53.0	49.2	52.0	52.3	52.9	52.3	51.4	54.8	55.0
Education >12y (%)	37.06	26.73	32.25	26.11	45.35	29.94	42.23	12.57	25.14	21.93	23.85	24.89	29.67
Larger cities (%)	38.19	34.71	35.90	37.79	29.58	36.71	33.47	32.41	32.92	42.88	35.00	29.90	30.95
Not born in Sweden (%)	14.28	14.72	14.63	17.68	11.12	12.84	12.04	17.70	17.02	21.34	18.16	14.08	13.96
Married or cohabitant (%)	54.14	56.50	59.14	57.95	54.70	62.89	56.49	34.75	45.34	38.25	41.63	55.43	52.62
Days hospitalized 2000-2005	1.4	6.2	3.6	3.9	3.7	4.0	2.2	28.6	5.2	7.6	5.6	5.4	5.2
Specialized outpatient clinics 2000-2005													
Circulatory	1.7	2.0	2.0	2.0	2.0.	2.1	2.0	2.0	2.0	2.0	2.0	2.1	2.0
Cancer	1.7	2.0	2.0	2.0	2.1	2.1	2.1	2.0	2.0	2.1	2.0	2.1	2.1

Note: All values other than for age and sex have been directly standardized according to the age distribution of the cohort. A number of individuals were included in more than one category.

^a^The reference group includes individuals without prevalent or incident sick leave or disability pension during 2005.

^b^ICD10: H60-H75, H80, H92, H94, H95.

^c^H83, H90, H91.

^d^H81, H82.

^e^H93.

Overall, we found no association between sick leave due to otoaudiological diagnosis and premature death in the crude analysis. After adjustment for potential confounders, such as age, sex, family situation, type of living area, country of birth, and days hospitalized, individuals with sick leave due to an otoaudiological diagnosis showed a lower mortality risk. Individuals on disability pension due to an otoaudiological diagnosis showed, however, an increased risk, HR 1.14 (95% CI = 1.01-1.29), in the multivariable analyses of all-cause mortality, than individuals not on sick leave or disability pension (Table 2). Analyses of the otoaudiological subcategories revealed the risk increase to be attributable to otological (HR 1.56; 95% CI = 1.04-2.33) and hearing (HR 1.20; 95% CI = 1.00-1.43) diagnoses.

**Table 2:** Hazard ratios (HRs) for all-cause mortality among individuals with prevalent sickness absence or disability pension due to an otoaudiological diagnosis in 2005

**Variable**	**Number of deaths**	**Crude HR (95% CI)**	**Age- and sex- adjusted HR (95% CI)**^**a**^	**Multivariable HR (95% CI)**^**b**^	**Multivariable HR including hospitalization (95% CI)**^**c**^
No sick-leave or disability pension	30 066	1.00 (ref)	1.00 (ref)	1.00 (ref)	1.00 (ref)
Sick leave					
With non-otoaudiological diagnoses	16 443	2.15 (2.11-2.19)	2.06 (2.02-2.10)	2.06 (2.02-2.10)	1.53 (1.50-1.56)
With otoaudiological diagnoses	67	0.99 (0.77-1.26)	0.85 (0.66-1.08)	0.90 (0.70-1.14)	0.75 (0.59-0.95)
Otological^d^	16	1.03 (0.63-1.69)	0.97 (0.60-1.59)	0.99 (0.61-1.62)	0.73 (0.45-1.19)
Hearing^e^	19	1.13 (0.72-1.80)	0.97 (0.61-1.54)	1.06 (0.67-1.67)	1.00 (0.63-1.58)
Vertigo^f^	20	1.01 (0.65-1.58)	0.87 (0.56-1.35)	0.90 (0.58-1.41)	0.68 (0.44-1.06)
Tinnitus^g^	13	0.88 (0.51-1.58)	0.70 (0.41-1.22)	0.74 (0.43-1.29)	0.71 (0.41-1.23)
Disability pension					
Non-otoaudiological diagnoses	27 204	6.44 (6.34-6.54)	3.94 (3.88-4.00)	3.46 (3.41-3.52)	2.45 (2.41-2.49)
Otoaudiological diagnoses	272	2.17 (1.93-2.46)	1.28 (1.13-1.45)	1.30 (1.15-1.47)	1.14 (1.01-1.29)
Otological^d^	25	2.40 (1.61-3.58)	1.89 (1.27-2.81)	1.80 (1.20-2.69)	1.56 (1.04-2.33)
Hearing^e^	139	1.75 (1.45-2.11)	1.29 (1.07-1.54)	1.31 (1.10-1.57)	1.20 (1.00-1.43)
Vertigo^f^	48	2.08 (1.56-2.78)	1.33 (1.00-1.77)	1.28 (0.96-1.70)	1.05 (0.79-1.43)
Tinnitus^g^	102	1.46 (1.18-1.80)	0.91 (0.74-1.12)	0.95 (0.77-1.16)	0.89 (0.73-1.10)

Note: A number of individuals were included in more than one category.

^a^Age (20–34, 35–44, 45–54, 55–64); sex (female, male).

^b^Additional adjustments: family situation (married/cohabitant, married/cohabitant with children, single, single with child, child living with parent); type of living area (larger cities, medium-sized cities, smaller places); country of birth (Sweden, Nordic countries, Europe, other part of the world); education (0–9, 10-12, >12 years), including 5 196 998 observations due to missing data on education and/or family situation.

^c^Additional adjustment; hospitalization days (total days 2000–2005: 0, 1–4, >4), including 5 196 998 observations due to missing data on education and/or family situation.

^d^ICD10H60-H75, H80, H92, H94, H95.

^e^H83, H90, H91.

^f^H81, H82.

^g^H93.

Disability pension due to vertigo or tinnitus was not associated with an increased mortality risk. In analyses of cause-specific mortality, however, we observed disability pension due to otoaudiological diagnoses to be positively associated with death from circulatory causes (Table 3).

**Table 3:** Hazard ratios (HRs) for cause-specific death among individuals with prevalent sickness absence or disability pension due to an otoaudiological diagnosis in 2005, with follow-up through 2010

***Cause of death***	**No. deaths**	**Crude HR (95%CI)**	**Age- and sex- adjusted HR (95% CI)**^**a**^	**Multivariable HR (95% CI)**^**b**^	**Multivariable HR including hospitalization HR (95% CI)**^**c**^
*Circulatory cause*					
No sick leave & no disability pension	7 018	1.00 (ref)	1.00 (ref)	1.00 (ref)	1.00 (ref)
All other sick-leave diagnoses	2 822	1.49 (1.43-1.55)	1.48 (1.43-1.55)	1.46 (1.41-1.53)	1.09 (1.04-1.13)
All sick leave with otoaudiological diagnoses	15	0.83 (0.50-1.40)	0.73 (0.44-1.23)	0.79 (0.47-1.32)	0.67 (0.40-1.13)
All other disability pension	7 421	8.13 (7.88-8.38)	4.66 (4.52-4.81)	4.00 (3.87-4.13)	2.81 (2.71-2.91)
All disability pension with otoaudiological diagnoses	70	2.79 (2.19-3.54)	1.49 (1.17-1.89)	1.50 (1.18-1.91)	1.28 (1.01-1.63)
*Cancer cause*					
No sick leave & no disability pension	12 916	1.00 (ref)	1.00 (ref)	1.00 (ref)	1.00 (ref)
All other sick-leave diagnoses	8 792	3.06 (2.98-3.14)	2.63 (2.56-2.70)	2.61 (2.55-2.68)	1.70 (1.65-1.75)
All sick leave with otoaudiological diagnoses	34	1.31 (0.93-1.85)	1.00 (0.71-1.41)	1.03 (0.73-1.44)	0.83 (0.59-1.17)
All other disability pension	7 930	4.14 (4.03-4.24)	2.08 (2.03-2.14)	1.97 (1.92-2.02)	1.41 (1.37-1.45)
All disability pension with otoaudiological diagnoses	124	1.99 (1.66-2.38)	1.00 (0.83-1.19)	1.02 (0.85-1.22)	0.84 (0.71-1.01)

Note: A number of individuals were included in more than one category.

^a^Age (20–34, 35–44, 45–54, 55–64); sex (female, male).

^b^Additional adjustments: family situation (married/cohabitant, married/cohabitant with children, single, single with child, child living with parent); type of living area (larger cities, medium-sized cities, smaller places); country of birth (Sweden, Nordic countries, Europe, other part of the world); education (0–9, 10-12, >12 years), including 5 196 998 observations due to missing data on education and/or family situation.

^c^Additional adjustments: hospitalization days (total days 2000–2005: 0, 1–4, >4); hospitalization days in specialized out-patient care in the same diagnostic chapter, including 5 196 998 observations due to missing data on education and/or family situation.

When stratifying the analyses of all-cause mortality by age, we observed a higher risk of premature death among individuals on disability pension due to otoaudiological diagnoses among individuals 57 years-old or younger than among those older than 57 (HR 1.39; 95% CI 1.11-1.76, compared with 1.15; 95% CI 1.00-1.33). When stratifying by sex, the following gender differences were observed: among women, neither sick leave nor disability pension due to an otoaudiological diagnosis was associated with mortality; among men, however, disability pension due to an otoaudiological diagnosis was associated with a 22% increased risk of death, but sick leave due to an otoaudiological diagnosis was inversely associated with mortality (Table 4).

**Table 4:** **Multivariable hazard ratios (HRs)**^**a **^**for all-cause death, stratified by age and sex, among individuals with prevalent sickness absence or disability pension due to an otoaudiological diagnosis in 2005, with follow-up through 2010**

	**Number of deaths**	**No sick leave & no disability pension**	**All sick leave with non-otoaudiological diagnoses**	**Sick leave with otoaudiological diagnoses**	**Disability pension with non-otoaudiological diagnoses**	**Disability pension with otoaudiological diagnoses**
Age						
≤57	37 157	1.00 (ref)	1.91 (1.86-1.96)	0.81 (0.55-1.21)	4.23 (4.13-4.34)	1.39 (1.11-1.76)
>57	33 789	1.00 (ref)	1.37 (1.34-1.41)	0.78 (0.57-1.06)	2.13 (2.08-2.18)	1.15 (1.00-1.33)
Sex						
Women	27 489	1.00 (ref)	1.51 (1.47-1.56)	0.87 (0.62-1.21)	2.24 (2.18-2.31)	1.02 (0.85-1.23)
Men	43 459	1.00 (ref)	1.55 (1.51-1.59)	0.64 (0.45-0.92)	2.56 (2.50-2.62)	1.22 (1.04-1.44)

Note: Mean age of death = 57 years.

^a^Age (20–34, 35–44, 45–54, 55–64) or sex (female, male), where applicable, and family situation (married/cohabitant, married/cohabitant with children, single, single with child, child living with parent); type of living area (larger cities, medium-sized cities, smaller places); birth country (Sweden, Nordic countries, Europe, other part of the world); education (0–9, 10-12, >12 years); hospitalization days (total days 2000–2005: 0, 1–4, >4), including 5 196 998 observations due to missing data on education and/or family situation.

## Discussion

In this population-based cohort, which included all individuals of working age (20–64 years), living in Sweden and not receiving an old-age or disability pension in 2005, we found an increased risk of premature death among those on disability pension with otoaudiological difficulties, which was attributable to otological and hearing diagnoses, compared with individuals without sick leave or disability pension reimbursed by Sweden’s Social Insurance Agency. The increased risk was more pronounced among younger individuals, and appeared to be confined to men and individuals with circulatory diseases. No increased risk of premature death was observed among individuals on sick leave due to an otoaudiological diagnosis compared with those without sick leave or disability pension.

Both all-cause sick leave and all-cause disability pension have previously been associated with increased mortality [7-18]. Previous studies of sick leave (all causes) and risk of death have, in general, shown an approximately three-fold increased risk of mortality, which is confirmed in this study in the category of sick leave due to other than otoaudiological diagnoses. Studies of all-cause disability pension have also shown, in general, an approximately three-fold increased risk of mortality, as does this study in the category of all disability pension due to diagnoses other than the otoaudiological. The considerably lower risk increases observed in this study support the notion that the implications of sick leave or disability pension are highly dependent on specific diagnoses.

This is the first study to assess mortality risk in relation to sick leave and disability pension due to otoaudiological diagnoses. Although hearing difficulties and other ear-related diagnoses are very common health issues, which affect the individual’s quality of life and work capacity, this is not believed to influence mortality to any significant extent. Some patients are operated on for middle-ear diseases or vestibular schwannomas, or are equipped with cochlear implants because of profound bilateral hearing loss, but the mortality rates attached to these procedures are very low and cannot explain our findings. Although hearing difficulties are not primarily something that directly cause death, one relevant hypothesis is that they lead to increased levels of stress, arising, for example, from constantly straining to hear [25]. Furthermore, there are likely comorbidities of hearing difficulties and other disorders. There are, for example, numerous scientific studies in which correlations between cardiovascular disease (CVD) or CVD risk factors and hearing loss have been reported [26,27]. It has even been suggested that hearing loss is an early marker of ischemic heart disease [27]. An interaction between auditory symptoms, CVD, and stress is also plausible. Moreover, the adverse effect of long-term absence from the labor market has been reported to influence the financial, social and family situation, leisure activities, sleep, psychological well-being, and lifestyle [28]. As in all studies of sick leave or disability pension due to specific diagnoses, the results are not directly translatable to all who are affected. Studies of sick-leave or disability pension diagnoses attribute to social consequences of disorders rather than morbidity [29,30]. Thus we aimed to study the combined effect of the vulnerability entailed by morbidity and the consequences of sick leave or disability pension.

Major strengths of the study are its population-based design, which includes the entire Swedish population of working age, and its complete follow-up. The study’s prospective nature and having register-based information of high quality make it highly unlikely that the results we observed were due to selection bias. Moreover, in Sweden, the work participation rate is very high, particularly among women and older individuals (groups with more frequent sick leave and disability pension), which is advantageous when studying these factors. One limitation is that we had a relatively short follow-up period. The register-based data do not allow for control of different lifestyle factors that might affect the risk of premature death, and the possibility of uncontrolled or residual confounding cannot be dismissed. We have, however, adjusted for multiple potential confounders, including a crude measure of morbidity (in terms of hospitalization days during the preceding years). Even though we included the whole Swedish population, several subgroup analyses, of, for example, rare causes of death such as suicide or traffic injury, were not possible due to low statistical power, although they would have been of interest.

## Conclusion

In conclusion, this nationwide cohort study suggests that sick leave due to otoaudiological diagnoses is not associated with increased mortality. However, disability pension due to otoaudiological diagnoses was found to be associated with a statistically significant increase in mortality risk in the analysis where adjustments were made for several potential confounders.

## Competing interests

The authors declare that they have no competing interests.

## Authors’ contributions

EF had full access to all the data used in the study and takes responsibility for the integrity of the data and the accuracy of the data analysis. EF also participated in the study design, did the statistical analyses, interpreted the data, and drafted the manuscript. UR participated in interpretation of the data and critical revision of the manuscript for important intellectual content. KA participated in the design, interpretation and critical revisions of the manuscript, and also obtained funding for the study. All the authors read and approved the final manuscript.

## Pre-publication history

The pre-publication history for this paper can be accessed here:

http://www.biomedcentral.com/1471-2458/14/137/prepub
